# Water, sanitation and hygiene in national action plans for antimicrobial resistance

**DOI:** 10.2471/BLT.20.284232

**Published:** 2021-06-01

**Authors:** Sabiha Essack

**Affiliations:** a Antimicrobial Research Unit, College of Health Sciences, University of KwaZulu-Natal, Private Bag X54001, Durban 4000, South Africa.

Antimicrobial resistance is a public health threat; global and national action plans that address this challenge should therefore be fit for purpose. Such plans should focus on preventing infectious diseases and sustaining the efficacy of antimicrobials. Preventing infection by implementing hygiene practices in everyday life settings reduces the need for antimicrobials and therefore the selection pressure for the development of antimicrobial resistance. Drug-resistant infections cause more serious illness, requiring complex and costly alternative treatments, and have higher mortality rates.^1^

The effectiveness of hygiene in lowering infection rates, reducing antimicrobial use and subsequent resistance is clear from a study in Cape Town, South Africa. The study assessed 685 households with children younger than five years in two separate geographical areas and found that hygiene education in combination with handwashing at critical times, regular bathing, disinfecting of household surfaces and proper waste disposal, resulted in up to five times fewer respiratory infections, and households were two and a half times less likely to experience gastrointestinal illness.^2^

The coronavirus disease 2019 (COVID-19) pandemic may contribute to reinforcing good hygiene habits, as public health authorities across the world have put an unprecedented focus on the role of hygiene in preventing the spread of the virus throughout the pandemic. Handwashing and disinfecting initiatives to mitigate the spread of the virus have been encouraged globally and, as people return to school and work, many countries have implemented stringent hygiene guidelines to prevent the risk of virus transmission.

## Existing action plans 

In 2015, the World Health Organization’s (WHO) Member States adopted the global action plan on antimicrobial resistance as the framework for combating antimicrobial resistance. This action plan makes 83 recommendations – covered under five main objectives – to Member States, the WHO Secretariat and other international and national stakeholders to tackle antimicrobial resistance. Infection prevention is one of the five strategies of the Global Action Plan on antimicrobial resistance. This strategy recommends stronger hygiene and infection-prevention measures, including vaccination, to limit the spread of resistant microorganisms and reduce antimicrobial use. Guidance, however, is focused primarily on infection prevention and control in health-care settings, with little or no focus on the importance of water, sanitation and hygiene as infection-prevention measures in homes and community settings.^3^

By 2021, nearly 140 countries have adapted this action plan to produce their own national action plans. Once the national plans have received official government approval, they appear on the WHO library and are publicly available. To date, 77 of the 140 existing plans are available in the WHO library.^4^

Here I discuss the omission of water, sanitation and hygiene strategies in the community in existing national action plans. This gap exists despite evidence to suggest the positive impact of such strategies on antimicrobial resistance. I advocate the inclusion of these strategies as an essential part of infection prevention and control recommendations.

## A policy challenge

Each year antimicrobial resistance causes 700  000 deaths worldwide.^5^ If this public health challenge is not mitigated, an estimated 10 million people will lose their lives to antimicrobial resistance by 2050. Approximately 9 million of these deaths are forecasted to occur in low- and middle-income countries in Africa and Asia,^5^ where infectious diseases remain the leading cause of death. Antibiotics have never been more vulnerable to drug-resistant bacteria and, with rates of resistance to some antibiotic–bacteria combinations rising to 70–90% in some low- and middle-income countries,^6^ sustained measures to contain antimicrobial resistance are needed.

## Omissions at community level 

One of the strategic objectives of the global action plan is to improve hygiene, sanitation and infection prevention and control to reduce the need for antimicrobials. National action plans recognize the importance of strengthening infection prevention in health-care settings with clear infection prevention and control strategies, but they give insufficient attention to such preventive measures in everyday life settings in the community.

Desktop research conducted to assess the recommendation of water, sanitation and hygiene strategies in the community as part of the infection prevention and control measures within the 77 national action plans in the WHO library^4^ revealed that only 11 of these action plans included such strategies (Box 1).

Box 1 **National action plans on antimicrobial resistance that include community-level water, sanitation and hygiene recommendations as part of their infection prevention and control strategy, by WHO Region and country**

**African Region**

Ethiopia: Expand infection prevention and control systems at national, regional and community levels. Develop infection prevention and control information tools, education, and promote behaviour change in communities, including schools and public gathering areas.

Mauritius: Review and update the national infection prevention and control policy and guidelines for infection prevention and control within communities.

South Africa: Include immunization programmes against preventable infections in infection prevention and control activities in the community and hospitals. Strengthen infection prevention and control in public health (water and sanitation).

**Region of the Americas**

Canada: Work with communities and stakeholders to build capacity and reduce inequalities in delivering comprehensive and effective infection prevention and control programmes in community settings.

United States: Enhance infection control within the community.

**Eastern Mediterranean Region**

Pakistan: Enable hygiene and sanitation at community level by providing and monitoring the quality of safe drinking water.

Sudan: Strengthen national capacity to provide safe water, sanitation and hygiene and conduct awareness campaigns on hygiene and safe water.

**European Region**

Turkmenistan: Raise awareness among patients about the simple measures to prevent and reduce infections in the family or community environment, such as handwashing and hygiene.

United Kingdom: Promote better infection prevention and control practices among the public by developing more targeted interventions to improve behaviour on hand hygiene.

**South-East Asia Region**

Democratic People's Republic of Korea: Promote healthy and hygienic behaviours at community level.

**Western Pacific Region**

Philippines: Promote and educate on infection prevention and control activities within the community.

WHO: World Health Organization.Data source: WHO, 2021.^4^

Most of the national action plans mention infection prevention and control in community settings in general terms, advocating for the promotion of and education on such activities within the community. None of the action plans specifically recommend the development of guidelines for infection prevention in the community, nor do they refer directly to hygiene and sanitation measures in everyday life settings. Only two of the WHO European Region’s plans (Turkmenistan and the United Kingdom of Great Britain and Northern Ireland) specifically address the nature of infection prevention and control activities. The British plan also acknowledges the need for targeted interventions to improve hygiene behaviour to ensure better infection prevention and control practices among the public.^4^

The national action plans of the Democratic People's Republic of Korea and the Philippines focus in general terms on the promotion of infection prevention and control activities and healthy and hygienic behaviours at community level with no mention of specific hygiene measures and interventions. Pakistan and Sudan’s national action plans recommend strengthening and promoting disease prevention practices in the community emphasizing the need for providing and monitoring the quality of safe drinking water, sanitation and hygiene.^4^

Canada and the United States of America are the only countries in the Region of the Americas to recommend infection prevention and control in community settings.^6^

Although only seven plans in the WHO national action plan library are from the African Region, four of these recognize the importance of water, sanitation and hygiene programmes in the community for infection prevention (Box 1). The South African action plan specifically addresses community infection prevention and control as one of its key strategic objectives and has implemented interventions to mobilize communities with respect to basic infection prevention and hand hygiene. This action plan recognizes that access to safe water and sanitation and hygiene services are a critical part of ensuring good hygiene in the community to reduce the spread of disease. 

## The missing link

Notwithstanding the national action plans, antimicrobial consumption and resistance levels continue to increase and spread. Such spread is driving the increased prevalence of antimicrobial resistance in communities, and drug-resistant infections are now circulating as widely in the community as in hospital settings. For example, extended-spectrum β-lactamase producing Enterobacterales, initially described in hospital infections, are increasingly circulating in the community and are frequently introduced into hospitals from the community, rather than vice versa. Colonization with extended-spectrum β-lactamase producing bacteria is very high even among healthy people, with one study in south India reporting a faecal carriage rate of about one third. ^7^

Poor sanitation and poor personal hygiene lead to infections that promote extensive antibiotic use. Studies suggest that handwashing alone could prevent one quarter of diarrhoeal episodes^8^ and reduce risks of respiratory infections by 6–44%.^9^ Respiratory tract infections and acute diarrhoeal diseases account for most antibiotic prescriptions, which are often inappropriate.^10^ Improving hygiene is thus an important factor in controlling antimicrobial resistance.

The home, schools and other everyday life settings provide multiple opportunities for the spread of infection, including the transmission of drug-resistant microorganisms within the community. These risks have recently been highlighted in a position paper developed by the Global Hygiene Council^11^ stressing the importance of the hands and contact surfaces within the home and other community settings as reservoirs of potentially infectious microorganisms. The importance of targeted hygiene within community settings is emphasized to reduce infection rates, the need for antimicrobials and the circulation of resistant bacteria within the community.

Lessons can be learnt from the implementation of community-level hygiene interventions that have proven successful in slowing the spread of severe acute respiratory syndrome coronavirus 2, the virus causing COVID-19. These water, sanitation and hygiene interventions are also essential to reducing the spread of other infections, including drug-resistant infections, with a resultant reduction in antimicrobial use and subsequent selection pressure for resistance. Preventing infection is an essential part of mitigating antimicrobial resistance, both in the community and health-care settings. National action plans due for review should ensure the inclusion of water, sanitation and hygiene community programmes. 
